# Supplementary material for: Reducing Aedes albopictus breeding sites through education: A study in urban area
Source: PLoS One. 2018 Nov 8;13(11):e0202451. doi: 10.1371/journal.pone.0202451 (PMC6224055; doi:10.1371/journal.pone.0202451)
Supplement: S1 File — The distributed brochure with general information about mosquitoes and their breeding sites. (PDF) [file pone.0202451.s001.pdf]

Κώνωψ ο σπιτικός

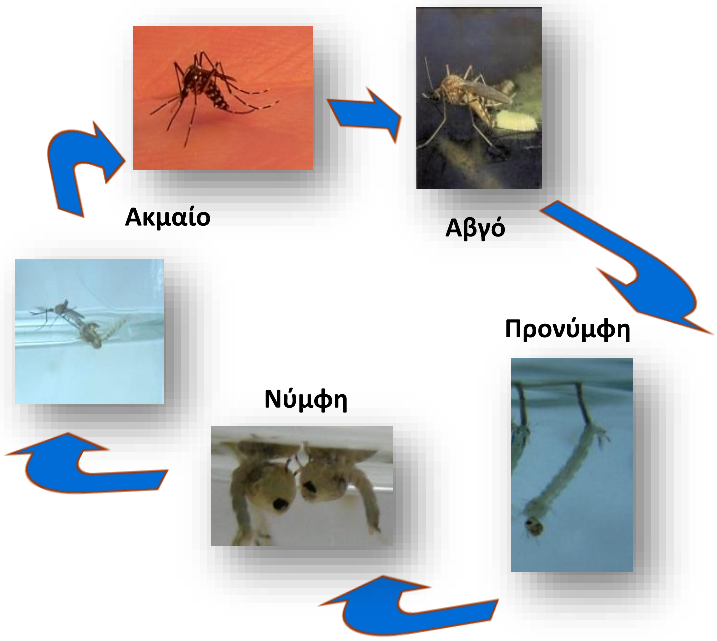

Ο **βιολογικός κύκλος του κουνουπιού** αποτελείται από το αβγό, τα προνυμφικά στάδια (προνύμφη), τη νύμφη και τέλος το ακμαίο. Εκτός από το τελευταίο στάδιο (ακμαίο-το γνωστό ενοχλητικό κουνούπι που μας τσιμπά) όλα τα άλλα διαβιούν στο νερό. Η διάρκεια του βιολογικού κύκλου των κουνουπιών εξαρτάται κυρίως από τη θερμοκρασία. Η διάρκεια της ζωής των ενηλίκων κουνουπιών (ακμαίων) εξαρτάται συνήθως από διάφορους παράγοντες όπως πχ θερμοκρασία, υγρασία, το φύλο του κουνουπιού και την εποχή του χρόνου. Τα αρσενικά ακμαία ζουν για περίπου μία εβδομάδα ενώ τα θηλυκά ζουν περισσότερο και ανάλογα με τους παραπάνω παράγοντες το διάστημα αυτό μπορεί να φτάσει έως και ένα μήνα. Τα κουνούπια, εκτός της όχλησης που προκαλούν, είναι οι φορείς αρκετών ασθενειών για τον άνθρωπο όπως ελονοσία (malaria), Δάγκειος αιμορραγικός πυρετός (dengue hemorrhagic fever), κίτρινος πυρετός (Yellow fever virus), τσικουνγκούνια (chikungunya) και ο ιός του Δυτικού Νείλου (West Nile virus).

Ποιες είναι όμως οι πιθανές εστίες στο σπίτι μας;

Γνωρίζοντας τις «συνήθειες» των κουνουπιών θα μπορέσουμε να επέμβουμε εγκαίρως όταν είναι «εγκλωβισμένα» ως προνύμφες στο νερό αντί να κυνηγάμε τα ακμαία φτερωτά άτομα! Τέλος, καλό θα είναι να φροντίζουμε, όπου είναι δυνατό, και τον ευρύτερο χώρο γύρω από το σπίτι μας. Εστίες όπως αυτές που αναφέρονται παρακάτω μπορεί να βρίσκονται έξω από τα όρια του κήπου μας αλλά σίγουρα τα κουνούπια δεν γνωρίζουν από τίτλους ιδιοκτησίας και τελικώς μας επισκέπτονται «ακάλεστα» και χωρίς ιδιαίτερους ενδοιασμούς.

1. Το **νερό** σε **σιντριβάνια**, μεγάλα **ανθοδοχεία** ή άλλες παρόμοιες **διακοσμητικές κατασκευές** θα πρέπει να αντικαθίσταται τουλάχιστον μια φορά την εβδομάδα. Τα **πιατάκια** επίσης **από τις γλάστρες** θα πρέπει να αδειάζουν σε τακτά χρονικά διαστήματα.
2. Πολλές φορές οι σχάρες των φρεατίων αποχέτευσης φράζουν από πεσμένα φύλλα, κομμένο γρασίδι ή άλλα σκουπίδια του κήπου με αποτέλεσμα να **παρεμποδίζεται η ροή του νερού** προς το δίκτυο αποχέτευσης.
3. Μικρές ή μεγάλες **λακκούβες στο χλοοτάπητα** ή σε άλλα σημεία του κήπου που μπορούν να συγκρατήσουν νερό θα πρέπει να παραγεμιστούν με χώμα ή να φροντίσουμε ώστε να αποστραγγίζουν καλά.
4. Διατηρείστε τα **λούκια και τις υδρορροές** στη σκεπή καθαρά καθώς εύκολα φράζουν από φύλλα ή κλαδάκια δένδρων και συγκρατούν το νερό της βροχής.
5. **Βρύσες** που στάζουν, αυτόματα ποτίσματα ή άλλες υδραυλικές εγκαταστάσεις με **διαρροές** μπορούν εύκολα να δημιουργήσουν μικρές εστίες ανάπτυξης κουνουπιών.
6. Τεχνητές λιμνούλες ή οποιαδήποτε φυσική ή μη **συγκέντρωση στάσιμου νερού** αποτελούν ιδανικές εστίες ανάπτυξης των εντόμων αυτών.
7. Ανοιχτά ή σπασμένα παράθυρα, φεγγίτες ή οπές εξαερισμού προσφέρουν εύκολους οδούς εισόδου των κουνουπιών προς το εσωτερικό των σπιτιών. Το **κλείσιμο των ανοιγμάτων** αυτών **με λεπτή ανθεκτική σήτα** είναι απαραίτητο.

Πηγή: www.conops.gr

8. Οι **πισίνες** θα πρέπει να αδειάζουν όταν δεν χρησιμοποιούνται καθώς και το νερό που πολλές φορές κατακρατείται από τα **καλύμματα** με τα οποία σκεπάζονται.

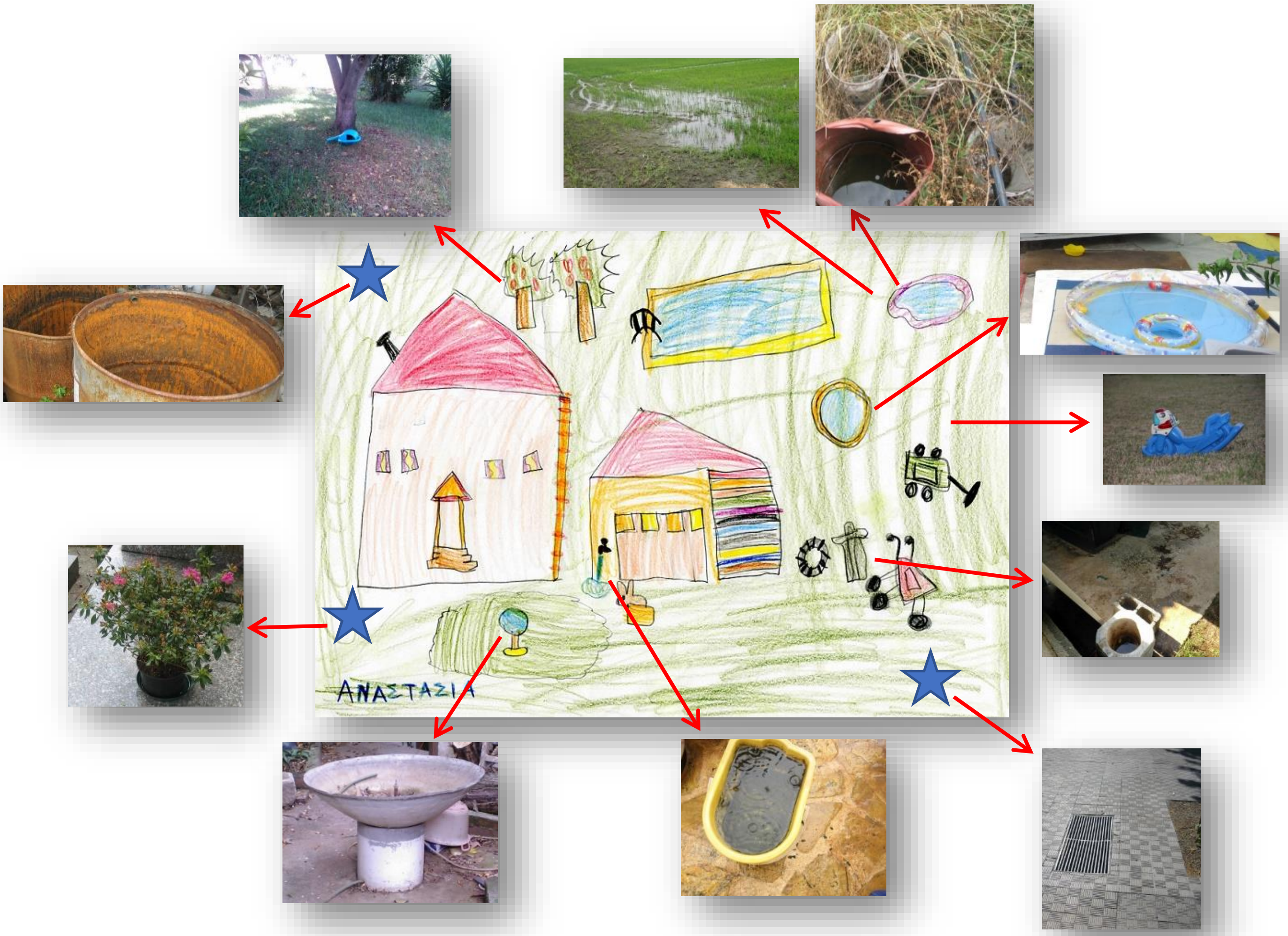

9. Παιδικές πισίνες, κουβαδάκια ή άλλα **παιχνίδια** που συνήθως αφήνονται μόνιμα στην αυλή ή στον κήπο θα πρέπει να τοποθετούνται σε κατάλληλα σημεία ώστε **να μην μαζεύουν το νερό της βροχής**.
10. Τα κουνούπια συχνά επιλέγουν να γεννήσουν τα ωά τους στο νερό που συγκεντρώνεται σε **δοχεία, βαρέλια, παλιά λάστιχα αυτοκινήτων** ή ακόμη και σε μικρά τενεκεδένια κουτιά που πολλές φορές υπάρχουν ξεχασμένα σε κάποια γωνία του κήπου. Για όσα από αυτά δεν μπορούν **να απομακρυνθούν** θα πρέπει να φροντίσουμε ώστε **να μην παραμένει στάσιμο νερό** στο εσωτερικό τους ανοίγοντας ίσως τρύπες στον πυθμένα τους ή σκεπάζοντάς τα.

ΜΠΕΝΑΚΕΙΟ ΦΥΤΟΠΑΘΟΛΟΓΙΚΟ ΙΝΣΤΙΤΟΥΤΟ

ΔΗΜΟΣ ΠΑΛΑΙΟΥ ΦΑΛΗΡΟΥ

Πηγή: www.conops.gr
