# Supplementary material for: Reducing Aedes albopictus breeding sites through education: A study in urban area
Source: PLoS One. 2018 Nov 8;13(11):e0202451. doi: 10.1371/journal.pone.0202451 (PMC6224055; doi:10.1371/journal.pone.0202451)
Supplement: S2 File — The distributed questionnaire (questions are translated in English in parenthesis). (PDF) [file pone.0202451.s002.pdf]

ΜΠΕΝΑΚΕΙΟ
ΦΥΤΟΠΑΘΟΛΟΓΙΚΟ
ΙΝΣΤΙΤΟΥΤΟ

Η ΕΛΑΙΑ ΤΟΥ ΠΛΑΤΩΝΟΣ

Τμήμα Εντομολογίας και Γ. Ζωολογίας
Μπενάκειο Φυτοπαθολογικό Ινστιτούτο
Στ. Δέλτα 8, 145 61 Κηφισιά
www.bpi.gr

Αποτίμηση των επιπτώσεων από τα κουνούπια στον Δήμο Παλαιού Φαλήρου

Το **Μπενάκειο Φυτοπαθολογικό Ινστιτούτο** προκειμένου να συμβάλει στον σχεδιασμό καταπολέμησης κουνουπιών πραγματοποιεί έρευνα με στόχο την αποτίμηση των κοινωνικοοικονομικών επιπτώσεων από τα κουνούπια στον Δήμο Παλαιού Φαλήρου.

Η παρούσα έρευνα έχει δομηθεί σε συνεργασία με το Cary Institute of Ecosystem Studies (με έδρα τη Νέα Υόρκη) και περιλαμβάνει το συγκεκριμένο ερωτηματολόγιο αλλά και τη διενέργεια επιτόπιας εντομολογικής έρευνας σε επιλεγμένες οικίες. Αναλυτικότερα:

- Στο πρώτο στάδιο, το ερωτηματολόγιο έχει ως στόχο την αποτύπωση βασικών πληροφοριών που σχετίζονται με τα κουνούπια και την κοινωνικοοικονομική κατάσταση των κατοίκων/ερωτώμενων

- Στο δεύτερο στάδιο της έρευνας, με βάση το ενδιαφέρον που θα δηλώσουν οι ερωτώμενοι, θα πραγματοποιηθεί εντομολογική έρευνα στις οικίες τους (επιλεγμένος αριθμός) και παράλληλα μια πιο εκτενής ενημέρωση σχετικά με τα κουνούπια και τη δημόσια υγεία στη γειτονιά σας.

Βοηθήστε εμάς, τη γειτονιά και τον Δήμο να ενημερωθούμε σχετικά με τα προβλήματα και τον έλεγχο κουνουπιών, απαντώντας στις παρακάτω ερωτήσεις. Η διάρκεια συμπλήρωσης του ερωτηματολογίου δεν ξεπερνά τα 5 με 8 λεπτά. Οι απαντήσεις είναι εμπιστευτικές και θα χρησιμοποιηθούν αποκλειστικά για ερευνητικούς σκοπούς.

Σας ευχαριστούμε εκ των προτέρων για τον χρόνο και τη συνεργασία σας.

Με εκτίμηση

Για το Μπενάκειο Φυτοπαθολογικό Ινστιτούτο	Για τον Δήμο Παλαιού Φαλήρου
Δρ Αντώνιος Μιχαηλάκης	Η Αντιδήμαρχος
	Περιβάλλοντος & Πρασίνου
Ερευνητής	Βασιλική Ανδρικοπούλου

Παρακαλούμε για την συμμετοχή σας και την συμπλήρωση του ερωτηματολογίου το αργότερο έως τις 27 Ιουνίου 2017 στην ηλεκτρονική διεύθυνση d.perivallon@palaiofaliro.gr

ΠΕΡΙΦΕΡΕΙΑ ΑΤΤΙΚΗΣ

Το παρόν ερωτηματολόγιο πραγματοποιείται στο πλαίσιο του έργου με τον τίτλο «Ερευνητικό Πρόγραμμα για την εκτίμηση των πληθυσμιακών διακυμάνσεων και την αξιολόγηση της υγειονομικής σημασίας των κουνουπιών στην Περιφέρεια Αττικής» που χρηματοδοτήθηκε από την Περιφέρεια Αττικής.

Τμήμα Εντομολογίας και Γ. Ζωολογίας
Μπενάκειο Φυτοπαθολογικό Ινστιτούτο
Στ. Δέλτα 8, 145 61 Κηφισιά
www.bpi.gr

☐

Βασικές ερωτήσεις (υποχρεωτικό πεδίο)

1. **Σε ποια περιοχή του Δήμου διαμένετε;** (What neighborhood do you live in?)

2. **Πόσο διάστημα διαμένετε στη συγκεκριμένη περιοχή;** (What neighborhood do you live in?)

3. **Εσείς ή κάποιο μέλος της οικογένειά σας χρησιμοποιεί κάποιο πάρκο ή κάποια πλατεία στη γειτονιά σας;** (Does anyone in your family use a park or a community garden in your neighborhood?)

   Ναι (YES) ☐ Όχι (NO) ☐

   Εάν ναι, πόσο συχνά και πώς ονομάζεται: (If yes, where and how often:)

4. **Ποιο νομίζετε ότι είναι το πιο σημαντικό περιβαλλοντικό πρόβλημα στη γειτονιά σας;** (What do you think are the most important environmental problems in your neighborhood?)

5. **Είστε ενήμερος/η σχετικά με τα αρθρόποδα στη γειτονιά σας (αρθρόποδα είναι για παράδειγμα τα κουνούπια, οι κοριοί, οι ψύλλοι, οι σκνίπες, τα ακάρεα, οι μύγες, οι**

ΠΕΡΙΦΕΡΕΙΑ ΑΤΤΙΚΗΣ
ΕΛΛΗΝΙΚΗ ΔΗΜΟΚΡΑΤΙΑ ΠΕΡΙΦΕΡΕΙΑ ΑΤΤΙΚΗΣ
ΔΗΜΟΣ ΠΑΛΑΙΟΥ ΦΑΛΗΡΟΥ
Το παρόν ερωτηματολόγιο πραγματοποιείται στο πλαίσιο του έργου με τον τίτλο «Ερευνητικό Πρόγραμμα για την εκτίμηση των πληθυσμιακών διακυμάνσεων και την αξιολόγηση της υγειονομικής σημασίας των κουνουπιών στην Περιφέρεια Αττικής» που χρηματοδοτήθηκε από την Περιφέρεια Αττικής.
ΠΕΡΙΦΕΡΕΙΑ ΑΤΤΙΚΗΣ ΑΘΗΝΑ

?

κατσαρίδες κ.ά.); (Are you concerned about outdoor pests in your neighborhood (for example, feral cats, rats, insects)?)

Ναι (YES) ☐ Όχι (NO) ☐

6. **Έχετε ενόχληση από κουνούπια;** (Are you ever bothered by mosquitoes?)

Ναι (YES) ☐ Όχι (NO) ☐

Εάν Ναι, πόσο συχνά δέχεστε ενόχληση από κουνούπια τους καλοκαιρινούς μήνες; (If yes, how often are you bothered by mosquitoes in the summer?)

Ποτέ (Never) ☐ Μερικές μέρες της εβδομάδας (A few days a week) ☐ Μερικές μέρες του μήνα (A few days a month) ☐ Κάθε μέρα (Every day) ☐

Άλλο (παρακαλώ περιγράψτε): [Other (please describe)]

7. **Υπάρχουν κουνούπια στην οικία σας (εντός ή/και εκτός);** (Are there mosquitoes on your property?)

Ναι (YES) ☐ Όχι (NO) ☐

Εάν ναι, από πού νομίζετε ότι προέρχονται τα περισσότερα κουνούπια; (If yes, where do you think most mosquitoes on your property are coming from?)

Στον ακάλυπτο χώρο σας (Your backyard) ☐ Στον γειτονικό ακάλυπτο χώρο (Your neighbors' backyards) ☐ Σε υδατοσυλλογές (Storm drains) ☐ Σε φρεάτια (Catch basins) ☐ Σε Πάρκο/Πλατεία/ποτάμι πλησίον (Park/Square/River nearby) ☐

Άλλο (παρακαλώ περιγράψτε): [Other (please describe)]

8. **Επηρεάζουν τα κουνούπια τις δραστηριότητές σας σε διάφορους εξωτερικούς χώρους;** (Do mosquitoes alter your outdoor activities?)

Ναι (YES) ☐ Όχι (NO) ☐

Εάν ναι, πώς; (If yes, how?)

Αναγκάζεστε να παραμένετε σε κλειστούς χώρους (Stay indoors) ☐ Αποφεύγετε συγκεκριμένες τοποθεσίες (Avoid certain areas) ☐ Αποφεύγετε την επικοινωνία σε

ΠΕΡΙΦΕΡΕΙΑ ΑΤΤΙΚΗΣ

Το παρόν ερωτηματολόγιο πραγματοποιείται στο πλαίσιο του έργου με τον τίτλο «Ερευνητικό Πρόγραμμα για την εκτίμηση των πληθυσμιακών διακυμάνσεων και την αξιολόγηση της υγειονομικής σημασίας των κουνουπιών στην Περιφέρεια Αττικής» που χρηματοδοτήθηκε από την Περιφέρεια Αττικής.

εξωτερικούς χώρους (Don't socialize outdoors) ☐ Δεν πηγαίνετε βόλτες με τα πόδια (Don't go for walks) ☐

Άλλο (παρακαλώ περιγράψτε): [Other (please describe)]

9. **Γνωρίζετε τα σημεία όπου τα κουνούπια εναποθέτουν τα αυγά τους και μεγαλώνουν;** (Where do the mosquitoes lay eggs and grow?)

   Ναι (YES) ☐ Όχι (NO) ☐

   Εάν Ναι, που; (If Yes, where?)

10. **Έχετε προβεί σε ενέργειες ώστε να μειώσετε τον πληθυσμό των κουνουπιών στην οικία σας;** (Do you do anything to keep the numbers of mosquitoes down on your property?)

    Ναι (YES) ☐ Όχι (NO) ☐

    Εάν Ναι, αναφέρατε ποιες. (If yes, what?)

11. **Ποιος νομίζετε ότι θα έπρεπε να είναι υπεύθυνος για την αντιμετώπιση των κουνουπιών;** (Who do you think should be responsible for mosquito control?)

    Το Υπουργείο Υγείας (Ministry of Health) ☐ Οι κάτοικοι (Residents) ☐ Οι ιδιοκτήτες των ακινήτων (Landlords) ☐

    Άλλο (παρακαλώ περιγράψτε): [Other (please describe)]

ΠΕΡΙΦΕΡΕΙΑ ΑΤΤΙΚΗΣ

Το παρόν ερωτηματολόγιο πραγματοποιείται στο πλαίσιο του έργου με τον τίτλο «Ερευνητικό Πρόγραμμα για την εκτίμηση των πληθυσμιακών διακυμάνσεων και την αξιολόγηση της υγειονομικής σημασίας των κουνουπιών στην Περιφέρεια Αττικής» που χρηματοδοτήθηκε από την Περιφέρεια Αττικής.

ΠΕΡΙΦΕΡΕΙΑ ΑΤΤΙΚΗΣ ΑΘΗΝΑ

12. **Στην κλίμακα από το 0 έως το 5, αναφέρατε πόσο σας απασχολεί η μετάδοση ασθενειών από τα κουνούπια;** (On a scale of 0-5, how concerned are you about diseases carried by mosquitoes?)

    (καμιά ανησυχία) 0 1 2 3 4 5 (πολύ μεγάλη ανησυχία)

    (Not at all concerned) (Very concerned)

13. **Γνωρίζετε τις ασθένειες που μπορούν να μεταδώσουν τα κουνούπια στην Αττική;**

    Ναι (YES) ☐ Όχι (NO) ☐

    Εάν ναι, αναφέρατε ποιες; (If Yes, which?)

14. **Γνωρίζετε ποια άλλα είδη ζώων μπορούν να νοσήσουν από τη μετάδοση ασθενειών από τα κουνούπια;** (What other animals can get diseases from mosquitoes?)

    Ναι (YES) ☐ Όχι (NO) ☐

    Εάν Ναι, αναφέρατε ποια; (If Yes, which?)

    Άλλο (παρακαλώ περιγράψτε): [Other (please describe)]

Δημογραφικά Στοιχεία (υποχρεωτικό πεδίο) [Demographic (obligatory data)]

Οι επόμενες ερωτήσεις αφορούν τη συλλογή βασικών δημογραφικών πληροφοριών.

1. **Πόσο χρονών είστε;** (How old are you?) 18-45 ☐ 46-60 ☐ +60☐
2. **Πόσο είναι το ετήσιο οικογενειακό σας εισόδημα;** (What is your household income?)

   ☐ λιγότερο από 5.000€ (5.000€ or less) ☐ από 5.000€ έως 10.000€ (5.000€ to 10.000€) ☐ από 10.000€ έως 20.000€ (10.000€ to 20.000€) ☐ από 20.000€ έως 30.000€ (20.000€ to 30.000€) ☐ άνω

ΔΗΜΟΣ ΠΑΛΑΙΟΥ ΦΑΛΗΡΟΥ

ΠΕΡΙΦΕΡΕΙΑ ΑΤΤΙΚΗΣ

Το παρόν ερωτηματολόγιο πραγματοποιείται στο πλαίσιο του έργου με τον τίτλο «Ερευνητικό Πρόγραμμα για την εκτίμηση των πληθυσμιακών διακυμάνσεων και την αξιολόγηση της υγειονομικής σημασίας των κουνουπιών στην Περιφέρεια Αττικής» που χρηματοδοτήθηκε από την Περιφέρεια Αττικής.

ΠΕΡΙΦΕΡΕΙΑ ΑΤΤΙΚΗΣ ΑΘΗΝΑ

30.000€ (more than 30.000€) □την παρούσα περίοδο είμαι άνεργος/η (I am currently unemployed)

3. **Σας ανήκει ή ενοικιάζετε το οίκημα που διαμένετε;** (Do you own or rent this property?)
   Ιδιοκτήτης (Owner) □ Ενοικιαστής (Tenant) □
4. **Έχετε παιδιά (ηλικίας <18) που διαμένουν μαζί σας;** (Do you have children (<18) living at this property?) Ναι (Yes) □ Όχι (No) □
5. **Ποιο είναι το επίπεδο της εκπαίδευσής σας;** (What is your educational level?)
   □Καμία τυπική εκπαίδευση/Βασική εκπαίδευση (Δημοτικό σχολείο)/Κατώτερη δευτεροβάθμια εκπαίδευση (Γυμνάσιο) [Less than Highschool]
   □Ανώτερη δευτεροβάθμια εκπαίδευση (Λύκειο)/Μεταδευτεροβάθμια εκπαίδευση μη Πανεπιστημιακή (ΙΕΚ) [Highschool/Post-Highschool studies]
   □ Πανεπιστημιακή εκπαίδευση (ΑΕΙ, ΤΕΙ) [University]
   □ Μεταπτυχιακή εκπαίδευση (MSc, PhD) [Post-graduate studies (MSc, PhD)]

Στοιχεία επικοινωνίας (προαιρετικό πεδίο) [Communication data (optional)]

Εφόσον επιθυμείτε να συμμετέχετε και στην επιτόπια έρευνα που θα πραγματοποιηθεί σε επιλεγμένα σπίτια καθώς και σε μια πιο εκτενή ενημέρωση σχετικά με τα κουνούπια και τη δημόσια υγεία στον Δήμο σας (γειτονιά σας) - παρακαλούμε να μας παρέχετε τα στοιχεία επικοινωνίας σας.

**Ονοματεπώνυμο:** (Name)	
**Τηλ. Επικοινωνίας:** (Tel.)	
**Email:**	

ΠΕΡΙΦΕΡΕΙΑ ΑΤΤΙΚΗΣ

Το παρόν ερωτηματολόγιο πραγματοποιείται στο πλαίσιο του έργου με τον τίτλο «Ερευνητικό Πρόγραμμα για την εκτίμηση των πληθυσμιακών διακυμάνσεων και την αξιολόγηση της υγειονομικής σημασίας των κουνουπιών στην Περιφέρεια Αττικής» που χρηματοδοτήθηκε από την Περιφέρεια Αττικής.
